# Therapeutic Targets in Allergic Conjunctivitis

**DOI:** 10.3390/ph15050547

**Published:** 2022-04-28

**Authors:** Bisant A. Labib, DeGaulle I. Chigbu

**Affiliations:** Pennsylvania College of Optometry, Salus University, Elkins Park, PA 19027, USA; dchigbu@salus.edu

**Keywords:** AC, CCR antagonist, JAK inhibitor, FAK inhibitor, integrin antagonist, RASP inhibitor

## Abstract

Allergic conjunctivitis (AC) is a common condition resulting from exposure to allergens such as pollen, animal dander, or mold. It is typically mediated by allergen-induced crosslinking of immunoglobulin E attached to receptors on primed conjunctival mast cells, which results in mast cell degranulation and histamine release, as well as the release of lipid mediators, cytokines, and chemokines. The clinical result is conjunctival hyperemia, tearing, intense itching, and chemosis. Refractory and chronic cases can result in ocular surface complications that may be vision threatening. Patients who experience even mild forms of this disease report an impact on their quality of life. Current treatment options range from non-pharmacologic therapies to ocular and systemic options. However, to adequately control AC, the use of multiple agents is often required. As such, a precise understanding of the immune mechanisms responsible for this ocular surface inflammation is needed to support ongoing research for potential therapeutic targets such as chemokine receptors, cytokine receptors, non-receptor tyrosine kinases, and integrins. This review utilized several published articles regarding the current therapeutic options to treat AC, as well as the pathological and immune mechanisms relevant to AC. This review will also focus on cellular and molecular targets in AC, with particular emphasis on potential therapeutic agents that can attenuate the pathology and immune mechanisms driven by cells, receptors, and molecules that participate in the immunopathogenesis and immunopathology of AC.

## 1. Introduction

Allergic conjunctivitis (AC) is an inflammation of the conjunctiva that involves immune mechanisms mediated by immunoglobulin E (IgE) and mast cells [1,2]. Although it affects up to 40% of people in the United States, AC is an often underdiagnosed, non-infectious subset of conjunctivitis. It results from exposure to allergens such as pollen, animal dander, and environmental stimuli [3,4]. Seasonal AC (SAC) and perennial AC (PAC) are the major subcategories of AC, with SAC making up approximately 90% of AC cases altogether. Other distinguished and less common forms include atopic keratoconjunctivitis (AKC) and vernal keratoconjunctivitis (VKC) [5].

Outdoor allergens, such as tree and grass pollen, are the most common stimuli of SAC, whereas indoor environmental allergens such as mold and animal dander are the main inciting agents in PAC [6,7,8]. In PAC, there is persistent allergen induced crosslinking of multiple IgE-FcεRI complexes on primed mast cells, leading to the persistent degranulation of mast cells and release of mediators that recruit eosinophils [1,9]. The most common feature associated with both SAC and PAC is the activation and degranulation of mast cells that release histamine, tryptase, prostaglandins, leukotrienes, cytokines, and chemokines which lead to the inflammatory response in AC [10]. Signs and symptoms of AC are variable and can be severe, impacting quality of life and can potentially be vision threatening. In the early stages of AC, when histamine binds with its receptor, clinical features such as itching, burning, tearing, chemosis, and papillae manifest. Later, immune cells, such as Th2 cells and eosinophils, invade the conjunctiva, leading to chronic inflammation. This has the potential to affect vision [11].

**Figure 1:**
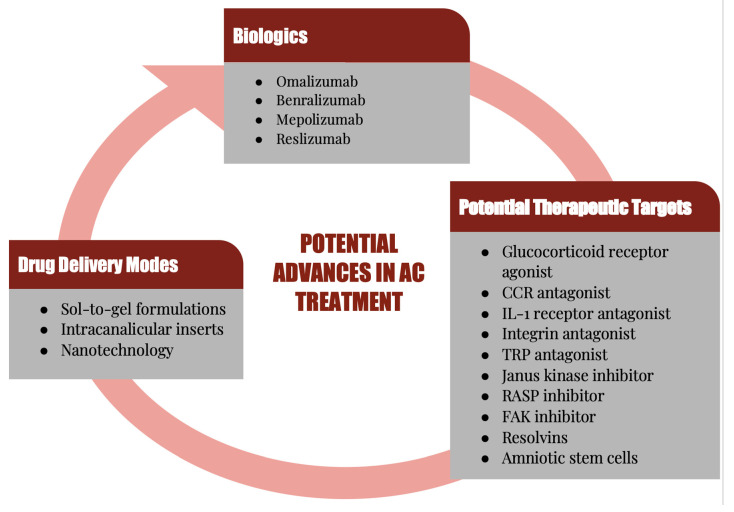
Summary of potential future therapeutic targets for allergic conjunctivitis. AC: allergic conjunctivitis; CCR antagonist: C-C chemokine receptor antagonist; L-1 receptor antagonist: Interleukin-1 receptor antagonist; TRP antagonist: Transient receptor membrane potential antagonist; RASP inhibitor: Reactive aldehyde species (RASP) inhibitor; FAK inhibitor: Focal adhesion kinase (FAK) inhibitor.

Although AC has always been a predominant form of conjunctivitis, it has increased in the last several decades, and is one of the most commonly encountered conditions by both allergists and eye care practitioners, affecting many patients and their quality of life [12,13,14,15]. Because of the high prevalence and the multifactorial causes of AC, a combination of therapies is often necessary to treat the associated signs and symptoms. This also creates a heavy economic burden, necessitating effective and novel treatment approaches, hence the ongoing research for additional therapeutic targets [10]. In this review, the cells and molecules that play a major role in the immunopathogenesis and immunopathology of AC will be highlighted. Current and potential future therapeutic agents that can prevent or treat the clinical expressions of AC via attenuation of the pathological and immune mechanisms of AC will be discussed (Figure 1).

## 2. Conjunctiva

The conjunctiva is a highly vascularized, immunologically active mucosal tissue that consists of an epithelium and stromal layer [16]. The conjunctiva is the port of entry for allergens such as pollen and animal dander. The conjunctival epithelial layer is a non-keratinized mucous membrane. The epithelial cells of the conjunctiva are held together by tight junctions, which in turn contributes to the physical barrier function of the conjunctiva that blocks access of allergens to primed mast cells and antigen-presenting cells in the conjunctival stroma. This is possible due to the presence of tight junctions that play a vital role in the maintenance of the epithelial barrier of the conjunctiva [17,18,19,20]. Goblet cells, suppressor CD8^+^T cells, cytotoxic CD8^+^T cells, melanocytes, and Langerhans cells are located in the conjunctival epithelium. The subepithelial layer of the conjunctiva consists of collagen, lymphoid follicles, fibroblast, vasculature, lymphocytes, macrophages, dendritic cells (DCs), and mast cells [1,21,22,23].

The conjunctival-associated lymphoid tissue (CALT) is an ocular surface immune protection system that consists of conjunctival lymphoid follicles (CLF) interspersed within the diffuse lymphoid effector tissue. The CLF constitutes the afferent arm of the CALT, and it consists of mucosal B cells, parafollicular T cells, lymph vessels, high endothelial venules (HEV), and apical follicle-associated epithelium (FAE) with M cells. The diffuse lymphoid effector tissue is the efferent arm of the CALT, and it consists of DCs, mast cells, macrophages, IgA-secreting plasma cells, and effector T cells. CALT provides immunosurveillance for the ocular surface through its ability to detect antigens and generate effector immune cells in response to invasion of the ocular surface [1,23,24,25,26]. Langerhans cells, DCs, and macrophages are important activators of the immune system, and they participate in the innate immune surveillance system of the conjunctiva. These sentinel immune cells constitute an important first line of defense of the ocular surface when the physicochemical barrier is breached [27].

Epithelial cells, antigen presenting cells (APCs), and fibroblasts of the conjunctiva participate in pathological and immune mechanisms of AC, and the inflammatory mediators secreted by these cells are responsible for the allergic inflammatory process in the conjunctiva [1,22]. Activated epithelial cells secrete inflammatory mediators such as cytokines, adhesion molecules, and chemokines that promote the infiltration of immune cells into the site of allergen-induced inflammation in the conjunctiva [22,28,29].

Conjunctival fibroblasts produce collagen. They also produce cytokines, adhesion molecules, and chemokines when they are activated. Fibroblasts can act as immune modulators in AC via their involvement in remodeling the conjunctiva in response to certain stimuli such as histamine derived from activated mast cells and cytokines derived from activated mast cells and Th2 cells [1,30,31,32,33]. Immune-mediated dysfunction of conjunctival fibroblasts, characterized by uncontrolled activation and proliferation of conjunctival fibroblasts, has been implicated in the development of conjunctival fibroproliferative lesions, such as papillae. Because epithelial cells and fibroblasts of the conjunctiva participate in the immunopathogenesis and immunopathological processes of AC, preventing inflammatory mediators from activating these cells can attenuate the pathological and immune mechanisms involved in AC [34]. As such, pharmacotherapeutic and immunotherapeutic agents that inhibit the effector function of these inflammatory mediators generated in response to allergens can provide therapeutic benefits for patients presenting with clinical expressions of AC.

Mast cells located in the subepithelial layer of the conjunctiva secrete both tryptase and chymase [20,35,36,37]. The cytoplasm of a mast cell contains up to 200 large granules with each granule containing histamine, heparin, tryptase, chymase, acid hydrolases, peroxidase, and phospholipases. Following the activation and subsequent degranulation of primed conjunctival mast cells, histamine and tryptase are released immediately, which is followed later by production of leukotriene B4 (LTB4), LTC4, prostaglandin E2 (PGE2), PGD2, platelet-activating factor (PAF), tumor necrosis factor alpha (TNFα), interleukin -1 (IL-1), IL-3, IL-4, IL-5, IL-9, IL-13, IL-25, stem cell factor (SCF), C-C motif chemokine ligand 2 (CCL2), CCL3, CCL5, CCL11, CCL17, vascular endothelial growth factor (VEGF), and nerve growth factor (NGF) [38,39,40].

## 3. Mediators of Allergy

Histamine is a biogenic amine that is responsible for the early clinical expressions of AC. Histamine mediates its effector function via the activation of the four types of histamine receptors, which are G protein-coupled receptors (GPCRs) [41,42]. The histamine type 1 (H1R) and type 2 (H2R) receptors are expressed on neurons, vascular smooth muscles, endothelial cells, epithelial cells, goblet cells, mast cells, neutrophils, eosinophils, monocytes, melanocytes, fibroblasts, macrophages, dendritic cells, T cells, and B cells [43,44,45,46,47]. H3 receptors are expressed on nerve cells and goblet cells [48], whereas H4 receptors are expressed on mast cells, fibroblasts, goblet cells, basophils, eosinophils, monocytes, macrophages, T cells, basophils, and dendritic cells [44,46,47,49]. H4 receptors mediate the recruitment and activation of mast cells [50], whereas histamine interacts with H4R expressed on eosinophils to mediate recruitment of eosinophils to the site of allergen-induced inflammation [51]. Papillary hypertrophy of the palpebral conjunctiva is due to histamine activation of conjunctival fibroblasts whereas ocular itch is due to histamine activation of conjunctival sensory nerve fibers. Histamine-induced dilation of conjunctival blood vessels manifests as conjunctival hyperemia, whereas increased blood flow and disruption of histamine-activated endothelial barrier function of the conjunctival blood vessels manifest as conjunctival chemosis. Activation of goblet cells is associated with mucus secretion that is likely to manifest as a watery mucoid discharge. Histamine can activate conjunctival epithelial cells to upregulate the expression of intercellular adhesion molecule 1 (ICAM-1), as well as secrete cytokines and chemokines [1,48,52]. Antagonists of histamine receptors can attenuate the effector function of histamine since biological effects of released histamine is mediated through histamine receptors (see Section 5.2). Histamine can also activate the transient receptor potential vanilloid 1 (TRPV1) receptor expressed on conjunctival sensory neurons and epithelium. It has been demonstrated that the activation of the TRPV1 receptor enhances histamine-dependent ocular itch [53,54].

Adhesion molecules such as integrins and intercellular adhesion molecules play a role in the pathological and immune mechanisms of AC. Integrins are heterodimeric cell adhesion receptors that consist of an alpha domain (CD11a) and a beta domain (CD18). Lymphocyte function associated antigen-1 (LFA-1), a member of the beta2 integrins, is expressed on immune cells including T cells and eosinophils. It promotes the interaction between cells or between cells and the extracellular matrix. LFA-1 interacts with intercellular adhesion molecules (ICAM), such as ICAM-1, expressed by vascular endothelial cells to promote the induction of microvascular dilatation and permeability as well as facilitate the adherence and subsequent transmigration of effector immune cells into the site of inflammation [55,56,57,58,59]. Intercellular adhesion molecule 1 (ICAM-1) is an adhesion molecule that belongs to the immunoglobulin superfamily. It is expressed on epithelial cells, endothelial cells, and fibroblasts. ICAM-1 can act as an inflammatory modulator that plays a role in mediating the interaction between immune cells and vascular endothelium, as well as facilitate the attachment of effector T cells to activated epithelial cells of the conjunctiva [56,58,59]. Thus, targeting integrins and focal adhesion kinase, a downstream mediator of integrins, [60] could be beneficial in attenuating cellular processes involved in AC.

Leukotrienes and prostaglandin are lipid mediators generated from arachidonic acid metabolism via enzymatic action of cyclooxygenase and lipoxygenase. It is noteworthy that phospholipase A2 catalyzes the release of arachidonic acid from membrane phospholipids [61]. Leukotriene is a lipid mediator that causes vasodilation and increased permeability of conjunctival blood vessels [61,62,63]. Prostaglandin has been shown to intensify the ocular itch and cause dilation of conjunctival blood vessels [64,65]. Cytokines are small, secreted cell-signaling proteins that are released by both immune and non-immune cells. They bind to their cognate receptors to induce autocrine or paracrine effects in both innate and adaptive immune systems. Cytokines can exhibit either pro-inflammatory or anti-inflammatory activities [66]. Cytokines such as IL-4 act on conjunctival fibroblasts to induce the development of papillary hypertrophy of the palpebral conjunctiva. IL-5 mediates the activation and recruitment of eosinophils, whereas IL-13 induces goblet cells located in the conjunctival epithelium to secrete mucus [1,34,67]. Interleukin cytokines, such as IL-4, IL-5, and IL-13, that participate in the pathological and immune mechanisms of AC, signal through type 1 cytokine receptors. Type 1 and type 2 cytokine receptors are the two primary types of cytokine receptors. Cytokine receptors are glycoproteins that consist of an extracellular domain that binds to cytokines and an intracellular domain that is responsible for signal transduction. The long cytoplasmic domain of the cytokine receptor has an intrinsic protein tyrosine kinase activity that participates in signal transduction. Cytokines bind to their receptors to induce a conformational change that brings cytoplasmic receptor associated Janus kinase (JAK), a member of the tyrosine kinases, into close proximity. The association of JAK with the cytoplasmic tail of the cytokine receptor results in the activation of associated cytoplasmic JAK, as well as the phosphorylation of tyrosine residues on the cytoplasmic domain of the cytokine receptor. The phosphorylated JAK becomes a docking site for signal transducer and activator of transcription (STAT) proteins. The recruitment and binding of STAT proteins to the activated JAKs results in the phosphorylation and dimerization of STAT proteins. Subsequently, the phosphorylated STAT proteins dissociate from the cytokine receptor and translocate to the nucleus, where they bind to target genes in order to induce the transcription of genes [68,69,70,71]. Cytokines bind to their receptors to mediate the activation of JAK/STAT pathways, a signaling pathway that is located downstream of type 1 and 2 cytokine receptors. Thus blocking the binding of a cytokine to its receptor will result in a lack of signaling due to the non-activation of JAK and STAT, and as such, there is no cytokine-mediated activity. Cytokine receptor antagonists prevent the induction of signaling pathways in the cytokine receptor, whereas inhibiting the action of cytokines attenuates cytokine-induced inflammation. Blocking the JAK/STAT pathway attenuates the downstream signaling cascade through the cytokine receptor.

Chemokines are small-sized chemoattractant cytokines secreted by immune and non-immune cells. They bind to G-protein coupled receptors (GPCR) with seven transmembrane domains expressed on the surface of immune cells, such as lymphocytes and eosinophils, to initiate a signaling cascade that induces migration of immune cells along a chemotactic gradient. As such, chemokines promote the migration of immune cells toward a chemokine secreting immune or non-immune cell [72,73]. Chemokines secreted by mast cells, activated epithelial cells, and activated fibroblasts promote the infiltration of immune cells to the site of allergen-induced conjunctival inflammation. C-C motif chemokine ligand 2 (CCL2) and CCL3 are secreted by fibroblasts and epithelial cells. C-C chemokine receptor 2 (CCR2) is a receptor for CCL2, whereas CCL3 is a ligand for CCR1 and CCR5. CCL2 interacts with CCR2 to mediate the migration of mast cells towards the site of inflammation [74], whereas CCL3 has been demonstrated to be capable of activating mast cells to release histamine [75]. CCL5 is a ligand for CCR1, CCR3, CCR4, and CCR5. It is secreted by endothelial cells, macrophages, mast cells, and fibroblasts. CCL5 mediates the recruitment and activation of eosinophils [76]. CCL7 is produced by fibroblasts, and it is a ligand for CCR1, CCR2, and CCR3. It is a potent chemotactic agent for recruiting eosinophils in mucosal type 2 immune responses [77], and it has been shown to induce mast cell activation [78]. Because CCL7 induces mast cell activation, Kuo et al. [78] suggested that chemokine-targeting therapy could be useful in attenuating type I hypersensitivity responses that are characteristic of allergen-induced inflammation of the conjunctiva. CCL11 is a ligand for CCR2, CCR3, and CCR5. CCL11 is secreted by epithelial cells and fibroblasts, and it mediates the recruitment of eosinophils. CCL17 is a ligand for CCR4. CCL17 is produced by epithelial cells, dendritic cells, and monocytes. CCL17 is a major chemotactic agent for T cells. CCR3 serves as a receptor for CCL24 and CCL26, whereas CCR2 serves as a receptor for CCL26. Eosinophils are recruited to the site of allergen-induced conjunctival inflammation by CCL24 and CCL26 [72,73,76]. It is important to note that signaling via CCR3, expressed on mast cells and eosinophils, is essential for CCR3-mediated activation and recruitment of mast cells and eosinophils. As such, inhibiting CCR3-mediated immune responses in AC could be therapeutically beneficial.

## 4. Pathological and Immune Mechanism

The immunopathogenesis of AC is initiated upon first exposure of the conjunctiva to environmental allergens. These allergens secrete proteases that activate protease activated receptors to initiate the disruption of the barrier function of the conjunctival epithelium. This barrier function disruption allows access of allergens to antigen-presenting cells (APC) in the conjunctival stroma. A critical step in the immunopathogenesis of AC is to generate Th2 cells that secrete IL-4, which in turn induces the differentiation of allergen-specific B cell into IgE-secreting plasma cells. The allergen-specific IgE binds to the immunoglobulin-like domain of alpha chain of Fc Epsilon Receptor I (FcεRI) on conjunctival mast cells, and this interaction results in the formation of a IgE-FcεRI complex on conjunctival mast cells. This results in the priming of the conjunctival mast cells. Another major pathway in the immunopathogenesis of AC involves generating thymic stromal lymphopoietin (TSLP) from conjunctival epithelial cells activated by environmental allergens. TSLP binds to TSLP receptors on conventional DCs, which primes DCs to activate naïve T cells in the paracortex of the regional lymph node, these activated T cells proliferate and differentiate into Th2 cells that produce IL-4. The cytokine produced, IL-4, interacts with IL-4 receptors on allergen-specific B cells inducing their proliferation and differentiation into IgE-secreting plasma cells. The allergen-specific IgE then binds to the immunoglobulin-like domain of alpha chain of FcεRI on mast cells within the stroma of the conjunctiva. It is important to note that the successful formation of the IgE-FcεRI complex leads to the priming of conjunctival mast cells. This process is the sensitization phase of the allergic immune response [1,79]. IgE is the main immune reactant of AC, a type I hypersensitivity immune response of the conjunctiva. As such, breaking up the IgE-FcεRI complex on conjunctival mast cells is therapeutically beneficial for patients with AC [80].

Re-exposure of primed conjunctival mast cells to pollen allows for pollen to induce crosslinking of polyvalent IgE-FcεRI complexes on primed conjunctival mast cells. This results in receptor aggregation that initiates a signaling cascade, which culminates in the activation of signaling proteins such as syk tyrosine kinase. This signaling protein phosphorylates linkers for activation of T cells (LAT). Phospholipase Cγ (PLCγ) is phosphorylated by LAT, and this initiates a downstream signaling cascade event that produces inositol-1,4,5-triphosphate (IP3) and diacylglycerol (DAG). IP3 causes the mobilization of calcium within the mast cell [81,82], this leads to activation and degranulation of primed conjunctival mast cells to release early phase (preformed) mediators such as histamine, as well as generate lipid mediators, cytokines, and chemokines in the late phase of the allergic response. The early and late phases of the allergic immune response constitutes the activation phase of the immune mechanisms of AC. This represents the immunopathological mechanisms of AC [1,42,43,44,49,80,83,84]. The infiltration of immune cells in the late phase of the allergic immune response occurs in PAC. Th2 cells and eosinophils are recruited by CCL17 and CCL11, respectively. IL-5 and ICAM-1 expressed by conjunctival epithelium promote the recruitment and infiltration of eosinophils into the conjunctiva. Histamine, leukotrienes, prostaglandins, cytokines, chemokines, and growth factors are mediators of the immunopathological mechanisms of AC, which culminates in the clinical expressions of the allergen-induced inflammation of the conjunctiva. Prophylactic and therapeutic modalities that target these mediators and inhibit their activity as well as attenuate the clinical expressions of allergen-induced inflammation will provide clinical benefits.

## 5. Current Treatment Approaches

### 5.1. Non-Pharmacologic Options

Treating this complex and multifactorial condition begins with removing the inciting allergen. This is difficult to do, as it is often in contact with a combination of allergens that produces this response. The ocular surface is also a largely exposed surface area, which does not allow for easy avoidance of environmental irritants [10]. Particularly, avoiding environmental allergens such as pollen or mold would require staying indoors with the windows closed, which is often impractical. Wearing large, protective sunglasses outdoors can help limit the ocular surface exposure to the surrounding environment [85]. The same challenge can be said for pet allergies; frequent bathing of the animal and limiting in-home carpeting to reduce pet dander is a considerable option [86]. Strategies for dust mite allergies include the use of HEPA (high-efficiency particulate air) filters and frequent laundering of bedding in high heat. Many of these strategies are not fully effective, and if so, are more useful for allergic rhinitis than for ocular signs and symptoms [87]. As such, first line treatments often include the use, or adjunctive use, of over-the-counter artificial tears to flush the ocular surface from inflammatory mediators [10,88,89]. Cold compresses or saline can also be used to relieve acute symptoms, or in combination with other therapies [90,91,92]. Applying cool compresses can help in the acute phase of AC, allowing for the superficial vessels to vasoconstrict and limit hyperemia. Oftentimes, however, both allergen avoidance and non-pharmacologic methods are inadequate to achieve long term, and sometimes even acute relief of symptoms [87].

### 5.2. Antihistamines

Anti-allergic therapeutic agents are subdivided to include antihistamines, mast cell stabilizers, or a combination. One of the most common current therapeutic approaches is blocking histamine receptors, thereby attenuating the allergen-induced inflammation of the conjunctiva. These may be administered either topically or systemic. Topical antihistamines work competitively to block histamine receptors, preventing the associated itching and hyperemia. However, the duration of action is limited in that it works for only a short period of time, necessitating frequent dosing and poor compliance. Additionally, other pro-inflammatory mediators that are typically released in an allergic response, such as prostaglandins and leukotrienes, are not impacted by antihistamines, therefore also limiting its use [11]. When administered in combination with topical decongestants, antihistamines have been shown to be more effective, but again, require frequent dosing at four times per day [93]. Topical decongestants are non-prescription options that work as alpha-adrenergic agonists to induce vasoconstriction of the superficial blood vessels in the conjunctiva to reduce conjunctival hyperemia and edema. Examples of these drugs are naphazoline, tetrahydrozoline, oxymetazoline, and brimonidine tartrate. Caution must be exercised with this combination, as decongestants are effective vasoconstrictors, but may lead to rebound hyperemia and tachyphylaxis with prolonged use. Additional adverse effects include stinging, mydriasis, and rebound conjunctival hyperemia. Antihistamines in and of themselves may also be irritating to the ocular surface with prolonged use [93,94,95]. Hence, while seemingly efficacious in the short term at relieving signs and symptoms, long term use of decongestants or combination antihistamine/decongestants is not very viable [11].

Antihistamines can also be used orally to treat symptoms associated with AC. The second-generation antihistamines are preferred, due to their higher safety profile. Using any type of oral antihistamine alone is not typically recommended, as a common side effect is the drying of the ocular surface, which may actually exacerbate symptoms of AC. As such, artificial tears or other forms of topical ocular treatments should be used in conjunction [10,96]. Examples include loratadine, desloratadine, and fexofenadine. More recently, known potent oral antihistamines, cetirizine and bilastine, have been formulated for ophthalmic use and are showing promise for AC treatment [97]. Bilastine, for example, is a well-established second-generation oral antihistamine commonly used in allergic disorders. An ophthalmic formulation of bilastine was more recently developed and is currently undergoing clinical trials to evaluate its efficacy when used topically for ocular allergy. A study evaluated preservative free 0.2%, 0.4%, and 0.6% ophthalmic formulations and showed superiority using 0.6%. This subset of treated patients reported improvement in ocular itching, tearing, edema, and conjunctival redness as early as 15 min following instillation and up to 16 h after that. Patients receiving this treatment reported good comfort with minimal, if any, adverse effects [98]. Levocabastine 0.05% ophthalmic suspension and emedastine 0.05% ophthalmic solution are other ophthalmic antihistamines that are dosed one drop four times daily in each eye to relieve the signs and symptoms of AC (Table 1) [99,100,101].

### 5.3. Mast Cell Stabilizers

Another current therapeutic target is mast cells. Mast cell stabilizers differ slightly in mode of action in that they decrease or inhibit mast cell degranulation. The precise mechanism is thought to be due to inhibition of calcium mobilization in the mast cell. This resultant inhibitory mechanism not only prevents or decreases mast cell degranulation, but further prevents release of histamine and other chemotactic factors [102]. The drawback to mast cell stabilizers is that they cannot work retroactively to offer acute symptomatic relief. Rather, they are best when used prophylactically to prevent future mast cell degranulation from allergen exposure. As such, using this class of medications topically, as a loading dose, and prior to allergen exposure, is the most effective method of administration [10]. Mast cell stabilizers such as lodoxamide tromethamine, pemirolast potassium, and sodium cromoglycate are usually dosed four times daily, except nedocromil that is dosed one drop two times daily (Table 1). Lodoxamide tromethamine 0.1% ophthalmic solution is safe for use in children and adults, and it is considered to be more potent than sodium cromolyn. Pemirolast potassium 0.1% ophthalmic solution is a mast cell stabilizer that is approved for the treatment of seasonal allergic in children as young as 3 years of age [95,100,103,104]. Nedocromil sodium 2% ophthalmic solution is an effective and well-tolerated mast cell stabilizer for long-term management of AC [105,106].

### 5.4. Combination Antihistamine/Mast Cell Stabilizers

To achieve optimal therapeutic benefit, combination antihistamine and mast cell stabilizers have become the mainstay for treatment when it comes to treatment of allergic disease including AC. The dual mechanism allows for both short- and long-term relief with a good safety profile [11,87]. Examples of these drugs include olopatadine (0.1% Patanol, 0.2% Pataday, 0.7% Pazeo, Novartis), ketotifen 0.025% (Zaditor^®^, Novartis), bepotastine besilate 1.5% (Bepreve^®^, Bausch & Lomb), epinastine 0.05%, alcaftadine 0.25%, and azelastine 0.05%. These ophthalmic antihistamine/mast cell stabilizer combination agents are usually dosed twice daily, except alcaftadine, Pataday, and Pazeo, which are dosed one drop once daily (Table 1) [8,11,107]. Many specific drugs in this class have additional mechanisms that mediate inflammation and target cells that are implicated in AC, such as eosinophils [11].

Ketotifen is a combination antihistamine and mast cell stabilizer, which also has the added benefit of eosinophil, leukotriene, and cytokine inhibition [108,109]. Azelastine is a second generation H1 receptor antagonist, but also inhibits platelet activating factor (PAF) and intercellular adhesion molecule 1 (ICAM-1) [110]. Epinastine is an H1 as well as H2 receptor antagonist, with the addition of mast cell stabilizing and anti-inflammatory effects [111]. These combination drugs are often used as first line treatment for allergic conjunctivitis [10].

Studies evaluating olopatadine concluded that it was effective in decreasing histamine levels in the tear film, leading to markedly improved itching, tearing, edema, and chemosis, even in the long term with continued use [112,113,114,115]. These effects are relatively similar across the dual acting agents [116,117,118,119]. The most recently developed drug in this class is bepotastine. Its distinguishing properties are that it has an increased susceptibility to bind to H1 receptors with increased bioavailability. Due to these features, bepotastine can work as early as 15 min, with relief lasting up to 8 h [120,121].

McCabe and McCabe compared bepotastine with olopatadine hydrochloride 0.2% and found that, when dosed similarly, bepotastine was more effective at relieving both ocular and nasal allergic symptoms. The safety profiles were also similar, though a higher subset of patients reported a slightly unfavorable taste with bepotastine [122]. Overall, this newer drug in a well-established class of anti-allergy medications shows promise [87].

### 5.5. Nonsteroidal Anti-Inflammatory Drugs

When these aforementioned therapeutic options fail to adequately quell the hypersensitivity response, NSAIDs are sometimes prescribed as additive agents. While not necessarily specific to the treatment of AC, this class of drugs acts on the inflammatory pathway by blocking cyclooxygenase and the subsequent release of prostaglandins, which is a key inflammatory mediator in IgE related diseases such as AC. Thus, it aids in relieving the associated itching and redness in AC with minimal side effects [10,90,123]. This class of medications is a good option to use in the short term, as they can improve symptoms of itching, redness, tearing, and foreign body sensation. With frequent and prolonged overuse, however, adverse effects such as corneal keratitis, ulceration, or perforation can occur [124]. Ketorolac tromethamine 0.5% ophthalmic suspension is an FDA-approved topical NSAID that is dosed with one drop four times daily for the relief of ocular itching associated with seasonal AC. It can also reduce conjunctival hyperemia (Table 1) [1,3].

### 5.6. Corticosteroids

In refractory cases or to control acute exacerbations, topical corticosteroids can be utilized. Due to the severe inflammatory response that is often associated with AC, corticosteroids are very efficacious in reducing symptomatology. Due to their potency, they are effective among all forms of AC. Corticosteroids downregulate inflammation in the conjunctiva by binding to cytosolic glucocorticoid receptors to form a glucocorticoid–glucocorticoid receptor (GC/GCR) complex, which is transported to the nucleus where it binds to GC response elements (GREs) to upregulate the generation of anti-inflammatory mediators that inhibit inflammation and downregulate the generation of pro-inflammatory mediators [125,126]. Corticosteroids have anti-inflammatory and anti-proliferative activities, as well have the ability to mediate immunosuppression. These effects are possible due to their ability to inhibit the infiltration of immune cells into the site of inflammation [126], as well as their ability to induce the apoptosis of eosinophils and lymphocytes [127]. Additionally, corticosteroids induce the production of lipocortin, a glucocorticoid-induced protein that inhibits the enzymatic action of phospholipase A2, an enzyme required for the release of arachidonic acid from membrane phospholipids [126,128]. Corticosteroids can also inhibit histidine decarboxylase, an enzyme required for histamine synthesis in mast cells [1,126,129]. Corticosteroids inhibit capillary vasodilation and vasopermeability at the site of conjunctival inflammation. Corticosteroids also block the proliferation of fibroblasts and the deposition of collagen in the inflamed conjunctiva [126]. Corticosteroids are both potent and efficacious, however their safety profile is a drawback, due to side effects involving secondary infection, delayed wound healing, development of cataracts, and increased intraocular pressure leading to glaucoma [14,126]. These adverse effects limit their long term use [10].

The most potent topical corticosteroids are those which are ketone-based, such as prednisolone acetate 1% (Pred Forte^®^, Allergan, Dublin, Ireland), prednisolone phosphate 1%, and dexamethasone 0.1%. The greater the potency, the higher risk of steroid induced complications. Thus, less potent, ester-based corticosteroids are preferred as they are more efficiently metabolized and known to carry less side effects. Examples of those commonly used in AC are loteprednol etabonate (0.2% Alrex^®^, 0.5% Lotemax^®^ suspension, Bausch & Lomb) [130,131,132]. Topical corticosteroids are usually dosed one drop four times daily. Although topical corticosteroids such as Alrex 0.2% are usually prescribed for cases of acute flare up in cases of AC, caution is still exercised with long term use of any steroid. As such, effective and safe therapeutic agents are continuously being evaluated for the treatment of AC (Table 1) [88,89,133].

Interestingly, intranasal steroids such as fluticasone and mometasone also have the ability to improve ocular symptoms even though they were primarily formulated for allergic rhinitis. The reason for this is likely due to the afferent and efferent ocular-nasal relationship, which could be why intranasal administration of corticosteroids are sometimes more effective than oral antihistamines when it comes to AC [87,134,135,136].

### 5.7. Immunomodulators

A steroid sparing therapeutic option is topical immunomodulators, such as cyclosporine A (CsA), as they target and suppress T lymphocyte proliferation, thereby reducing mast cell release of histamine and eosinophil recruitment [137]. Both CsA and tacrolimus, are effective and steroid sparing alternatives for the treatment of various forms of AC. These drugs are calcineurin inhibitors that are safe for long term topical use. Though they are not approved for the treatment of AC, a dosing regimen of twice per day is effective for many types of inflammatory ocular surface disorders (Table 1) [11]. It has been demonstrate that topical cyclosporine 0.05% administered one drop twice daily was effective in reducing the clinical expression of AC [138]. Hazarika and Singh [139] demonstrated that topical 0.03% tacrolimus eye ointment dosed twice daily was effective at resolving the signs and symptoms of AC. They suggested that it was an excellent steroidal-sparing therapy for patients with persistent flare up of their AC. Systemic forms are also available for refractory or severe disease [11].

**Table 1:** Current therapeutic classes for the treatment of allergic conjunctivitis.

Drug Class	Mechanism	Current and Potential Drug Examples and Dosage
**Antihistamines**	Block histamine receptors, thereby, preventing histamine from interacting with histamine receptors [11].	Levocabastine 0.05% ophthalmic suspension: one drop four times per day [99,100,101]. Emedastine 0.05% ophthalmic solution: one drop four times per day [99,100,101]. Bilastine 0.6% ophthalmic formulation: one drop per day [98].
**Mast cell stabilizers**	Inhibit mast cell degranulation through prevention of calcium mobilization in the mast cell [102].	Lodoxamide tromethamine 0.1% ophthalmic solution: one drop four times per day [95,100,103,104]. Pemirolast potassium 0.1% ophthalmic solution: one drop four times per day [95,100,103,104]. Nedocromil sodium 2% ophthalmic solution: one drop twice per day [95,100,103,104]. Sodium cromoglycate 4% ophthalmic solution: one drop four per day [95,100,103,104].
**Combination antihistamines and mast cell stabilizers**	Histamine receptor antagonist and prevent mast cell degranulation [11,87].	Olopatadine ophthalmic solution (0.1% Patanol, 0.2% Pataday, 0.7% Pazeo, Novartis, Basel, Switzerland): one to two drops per day [8,11,107]. Ketotifen 0.025% ophthalmic solution (Zaditor^®^, Novartis): one drop twice per day [8,11,107]. Bepotastine besilate 1.5% ophthalmic solution (Bepreve^®^, Bausch & Lomb, Laval, QC, Canada): one drop twice per day [8,11,107]. Epinastine 0.05% ophthalmic solution: one drop twice per day [8,11,107]. Alcaftadine 0.25% ophthalmic solution: one drop per day [8,11,107]. Azelastine 0.05% ophthalmic solution: one drop twice per day [8,11,107].
**NSAIDs**	Blocks cyclooxygenase in the inflammatory pathway which inhibits prostaglandin release [10,90,123].	Ketorolac tromethamine 0.5% ophthalmic suspension: one drop four times per day [1,3].
**Corticosteroids**	Controls inflammation by forming a GC/GCR complex, which is transported to the nucleus where it binds to GREs to downregulate the generation of pro-inflammatory mediators [125,126].	Loteprednol etabonate (0.2% Alrex^®^, 0.5% Lotemax^®^ suspension, Bausch & Lomb): one drop four times per day [130,131,132].
**Immunomodulators**	Blocks IL-2 production, thereby, suppressing IL-2 mediated proliferation of T lymphocytes [137].	Cyclosporine A (0.05%) ophthalmic emulsion: one drop twice per day [138]. Tacrolimus ophthalmic solution or ointment: twice per day [139].

CL: contact lens; NSAIDs: non-steroidal anti-inflammatory drugs; GC/GCR: glucocorticoid-glucocorticoid receptor; GREs: GC response elements; IL-2: interleukin 2.

### 5.8. Immunotherapy

Allergen-specific immunotherapy has long been used in chronic treatment in patients with allergic rhinoconjunctivitis. This works by gradually introducing increasing levels of the known allergen to achieve desensitization or immune tolerance. The cellular process involves downregulation of the Th2 cell mediated immune response and the upregulation of the effector function of regulatory T-cells [11]. The focus has historically been on nasal symptoms rather than ocular, but improvements have been documented in both. Immunotherapy works to increase specific IgG4 and IgA production to reduce seasonal increases in IgE for that allergen. This, in turn, is supposed to build a gradual tolerance to the specific allergen. Administration of the allergen is delivered through subcutaneous or sublingual routes. The sublingual route appears to be more effective against ocular symptoms. However, despite the route, the nasal symptoms are more often alleviated than ocular. Immunotherapy is also not always effective, due to the systemic immune response to the administered allergen and subsequent reactions [10,140,141,142].

## 6. Potential Therapeutic Targets

### 6.1. Glucocorticoid Receptor Agonists

A potential area for the development of treatment of AC are the glucocorticoids. They may be efficacious, as they possess the anti-inflammatory properties of steroids, but are less inclined to cause the adverse effects of raising intraocular pressure. These drugs target multiple cytokines and chemokines involved in AC, thus inhibiting eosinophil activity [3]. This is accomplished through transrepression, where the glucocorticoid receptor interacts with classes of activating protein 1 and nuclear factor-kappa B. This ultimately results in the inhibition of transcription and enhances the anti-inflammatory effects that are clinically seen with treatment (Table 2) [143]. An example, mapracorat, is a nonsteroidal selective glucocorticoid receptor that is currently being studied for ocular use. It selectively and effectively binds to human glucocorticoid receptors and an effective option for the treatment of AC, as it targets eosinophils, inhibiting migration and inducing spontaneous apoptosis [87,144]. Moreover, evidence suggests it inhibits chemokine release from several cells involved in the allergic response, including mast cells, conjunctival epithelial cells, fibroblasts, and corneal epithelial cells [145,146,147]. Unlike other glucocorticoids, mapracorat is not as effective in its ability to promote transactivation of genes, allowing for fewer ocular and systemic side effects than is normally seen with glucocorticoid receptors, while still remaining a good anti-inflammatory drug option [148]. Additionally, as it is a nonsteroidal agent, its likelihood of causing IOP elevation is markedly lower. This was confirmed in a study that compared it to dexamethasone, where mapracorat was shown to have less risk of raising IOP [149]. Further evidence suggests that mapracorat may also partially function to induce myocilin, a trabecular meshwork protein that is associated with IOP elevation, lessening the risk of ocular complications [143,150]. Experimental and ongoing clinical trials indicate that selective glucocorticoid receptor agonists may be an effective treatment option, as it combines both anti-inflammatory and immunosuppressive properties and limits adverse effects [11,151].

### 6.2. Receptor Antagonists

The often severe and rapid inflammatory response triggered in AC is the culmination of the release of mediators and recruitment of various inflammatory cells. Recruitment of immune cells occurs when chemokines bind to their cognate G-protein-coupled receptors (GPCRs) [152]. CCR3 is a type of GPCR implicated in both eosinophil and mast cell activation [153]. Kamatsu et al. studied the effects of this receptor in mice eyes, confirming its role in mast cell activation, vascular endothelial responses, and mRNA processing, further highlighting the impact of CCR3 on the allergic inflammatory response. As such, utilizing a CCR3 antagonist and anti-CCR3 antibody suppresses recruitment of eosinophils to the site of allergen-induced conjunctival inflammation and also inhibits CCR3-mediated activation of mast cells. As such, CCR3 antagonism can attenuate both early and late phase responses in AC (Table 2) [152].

Pollen in the tear film gains access to conjunctiva to induce multiple crosslinking of IgE bound to FcεRI on primed mast cells to trigger activation and degranulation of these mast cells. This results in the release of early phase mediators such as histamine that produce the clinical signs and symptoms seen in AC, as well as late phase mediators such as cytokines and chemokines that mediate infiltration of immune cells into the site of the conjunctiva. Chemokines such as CC chemokine ligand 11 (CCL11) are a key component in eosinophil and Th2 cell recruitment [154,155,156]. CCL11 levels, while normally present on the ocular surface in healthy eyes, are significantly elevated in AC [157,158,159]. Eperon et al. further concluded that IgE and CCL11 play a major role in the pathogenesis of SAC. IgE participated in the sensitization phase of the priming conjunctival mast cells as well as inducing mast cell degranulation and mediator release when pollen binds to IgE attached to FcεRI on primed conjunctival mast cells. CCL11 plays a role in recruiting eosinophils and Th2 cells that participate in the late and chronic phase of allergen-induced inflammation of the conjunctiva [160]. Since CC chemokines have been discovered to be a part of the late phase of the allergy reaction, a study using murine models deficient in CCL11 concluded that this CCR3 inhibition markedly reduces allergen-mediated hypersensitivity and IgE-mediated mast cell degranulation [160].

The CCL2/CCR2 interaction mediates recruitment of mast cell progenitors from circulation, toward the site of allergen-induced mast cell degranulation in order to replenish the degranulating mast cells. Blocking this interaction could be therapeutically beneficial since it reduces the recruitment and subsequent accumulation of mast cell progenitors that undergo terminal differentiation to become tissue-resident mast cells that express FCεRI. The terminal differentiation of mast cell progenitors requires the action of SCF and IL-3 at the site of allergen-induced inflammation in the conjunctiva [40,74,81,161]. To further investigate the role of CCR2 in AC, Tominga et al. discovered that mast cell-mediated acute inflammation is caused by CCL2/CCR2 interaction. Using murine mast cells and therapeutic interventions, they found that when the CCL2/CCR2 axis is inhibited, there was a reduction in both early mast cell activation and late-phase eosinophilic inflammation. These findings are important because they offer potential for therapeutic targets in AC (Table 2) [162].

Additionally, insunakinra (EBI-005) is an IL-1 receptor antagonist (IL-1Ra) that has been demonstrated to reduce inflammation of the ocular surface by blocking the IL-1 receptor [11,163]. IL-1 is a potent pro-inflammatory cytokine that mediates inflammation of the ocular surface by promoting the migration of immune cells into the site of inflammation in the conjunctiva. IL-1Ra inhibits the binding of IL-1 to the IL-1 receptor, causing the inhibition of IL-1-mediated inflammation in the conjunctiva [164] of a patient with AC (Table 2). Although Keane-Myers and colleagues [165] suggested that IL-1Ra can attenuate the influx of immune cells that facilitate the immunopathogenesis of allergic eye disease, further research in this area is warranted, especially in the specific context of AC.

Integrin antagonists, such as lifitegrast, is a lymphocyte function associated antigen-1 (LFA-1) antagonist that blocks the interaction between intercellular adhesion molecule-1 (ICAM-1) expressed on activated conjunctival epithelial cells and lymphocyte functional associated antigen 1 (LFA-1) expressed on Th2 cells. Blockage of ICAM-1 and LFA-1 interaction prevents the adhesion of Th2 cells to the conjunctiva, thus inhibiting Th2 cell mediated inflammation and release of Th2 cell derived cytokines (Table 2) [166]. Currently, it is an effective treatment option for ocular surface inflammation, mainly in patients with dry eye disease [11,166]. While integrin antagonist is not approved for treatment of AC, its impact on ocular surface inflammation generally may give it a place as a potential treatment or additive agent.

### 6.3. Transient Receptor Membrane Potential Antagonists

Stimuli receptors on the cell membrane, transient receptor potentials (TRP), include canonical, vanilloid, melastatin, mucolipin, and ankyrin subtypes. Both TRP ankyrin (TRPA1) and vanilloid (TRPV1) are involved in the increase of Th2 activity and related inflammation and have been implicated in allergic diseases such as allergic dermatitis and allergic rhinitis (Table 2) [167,168,169]. Their role in ocular allergy was evaluated by Kwon et al. using ovalbumin-sensitized AC murine models. In this study, topical administration of TRPA1 and TRPV1 antagonists were compared to determine the effect on signs and symptoms of AC. When compared to each other and to vehicle eye drops, the TRPV1 was the only group to have clinical improvement of AC and in symptoms of blinking and tearing. Furthermore, IgE levels in this group were also reduced. Although topical TRPA1 antagonist was shown to inhibit mast cell infiltration, it did not affect eosinophil infiltration, whereas TRPV1 antagonists not only inhibited both, but also attenuated IL-4 and IL-13 in conjunctival T cells. These results give rise to future promise [170].

**Table 2:** Potential therapeutic receptor agonists and antagonists in allergic conjunctivitis.

Receptor Agonists	Mechanism	Outcome
**Glucocorticoid receptor agonist**	Trans-repression through interaction with activating protein 1 and nuclear factor-kappa B [3,143].	Anti-inflammatory action with less potent side effects than steroids [143,150,151].
**Receptor Antagonists**	Mechanism	Outcome
**CCR3 antagonist**	Prevents mast cell activation and suppresses recruitment of eosinophils [152,160].	Suppression of clinical signs and symptoms in both early and late phase allergic responses [160].
**CCR2 antagonist**	Reduces recruitment and subsequent accumulation of mast cell progenitors [40,74,162].	Suppression of signs and symptoms in both early and late phase allergic responses [162].
**IL-1 receptor antagonist**	Blocks binding of IL-1 to IL-1 receptor, causing the inhibition of IL-1-mediated inflammation [164,165].	Reduces ocular surface inflammation [11,163].
**Integrin antagonist**	Blocks ICAM-1 and LFA-1 interaction which inhibits Th2 cell mediated inflammation [166].	Reduction in ocular surface inflammation, mainly in dry eye disease [11,166].
**Transient receptor membrane potential (TRP) antagonist**	Inhibits Th2 activity in allergic disease [167,168,169].	Clinical improvement in AC symptoms and reduced IgE levels [170].

CCR3: C-C chemokine receptor 2; CCR2: C-C chemokine receptor 3; IL-1 receptor antagonist: Interleukin-1 receptor antagonist; Th2 cell: T-helper 2 cell; AC: allergic conjunctivitis; IgE: immunoglobulin E; IL-1: interleukin 1; ICAM-1: intercellular adhesion molecule-1; LFA-1: lymphocyte function associated antigen-1.

### 6.4. Janus Kinase Inhibitors

Cytokines such as IL-4 and IL-5 play a major role in the immunopathology of AC, and as such, inhibition of the Janus kinase that associate with cytokine receptors can attenuate cytokine signaling required to induce immune-mediated conditions. A novel Janus kinase inhibitor, tofacitinib, has been documented to be an inhibitor of JAK1 and JAK3 that associate with cytokine receptors for IL-2, IL-4, IL-7, IL-9, IL-15, and IL-21. Inhibition of JAK3 reduces T cell proliferation mediated by IL-2, as well as blocks IL-4 mediated generation of Th2 cells (Table 3). It is also an inhibitor of JAK1-dependent STAT protein activation [171,172,173]. Tofacitinib has been documented to improve symptoms in patients with inflammatory autoimmune diseases such as psoriatic arthritis and ulcerative colitis [174,175]. Li et al. evaluated the efficacy of topical tofacitinib in experimental AC using mice models. Results were promising, in that there was significant improvement in itching, hyperemia and edema, in addition to a reduction in mast cell degranulation and inflammatory cytokine production [176]. Unlike the current mainstay of AC treatment, mast cell stabilizer/antihistamine combinations, tofacitinib is able to work on more targets, further reducing the inflammatory response through additional T cell, dendritic cell, and macrophage downregulation. While this drug shows promise for the treatment of AC, further studies are necessary [176].

### 6.5. Reactive Aldehyde Species Inhibitor

Reproxalap is a reactive aldehyde species (RASP) inhibitor that can be used topically to mitigate ocular inflammatory diseases such as noninfectious anterior uveitis and dry eye [177,178]. RASP is a known pro-inflammatory molecule that covalently binds to thiol and amino groups expressed on receptors and kinases to increase the pro-inflammatory signaling cascade that involves the activation of nuclear factor kappa B, inflammasomes, and prohistaminic factors. In periods of inflammation, such as AC, RASP can cause a sustained release proinflammatory cytokines and activation of inflammasomes (Table 3) [177,179,180,181,182]. By inhibiting this function and reducing the level of histamine and inflammation, signs and symptoms of AC can be attenuated. Clark et al. studied these findings using two concentrations of reproxalap ophthalmic formulations, 0.25% and 0.5%, in patients with AC by exposing them to allergen chambers and measuring treatment efficacy. The outcomes suggested the reproxalap was effective in both the prophylaxis of symptoms as well as treatment. A potential limitation is the adverse effect of stinging upon instillation, particularly with the 0.5% formulation [183]. The phase 3 ALLEVIATE trial investigated the effects of reproxolap on post-acute AC, defined as 10 min or longer following exposure of allergen to the conjunctiva. The results were promising, demonstrating safety and efficacy in both 0.25% and 0.5% concentrations. Patients in this study reported a marked improvement in ocular itching and tolerable use of reproxolap, indicating promise as a future treatment in AC [184].

### 6.6. Focal Adhesion Kinase Inhibitors

Focal adhesion kinase (FAK) is a non-receptor tyrosine kinase that regulates integrin-mediated signal transduction pathways, thereby playing a role in the control of cell migration, adherence, and proliferation [60,185]. FAK is also inhibitory against cellular apoptosis. Huang et al. [186] demonstrated that FAK facilitated cell survival and inhibited cytokine-mediated apoptosis via NF-kappa B pathway. Additionally, Sonoda and colleagues demonstrated that FAK has anti-apoptotic activity [187]. The interaction between integrins and their ligands induces the activation of FAK. The autophosphorylation of the activated FAK at tyrosine 397, results in the docking of Src family kinases to form an activated FAK/Src complex. The FAK/Src complex initiates a signaling cascade that regulates a wide range of biological activities such as proliferation, survival, and migration of cells [188,189]. FcεRI aggregation, affecting mast cell degranulation and related activity, has been shown to result in the autophosphorylation of FAK at tyrosine 397 in mast cells [190]. PF-431396 is both a FAK and proline-rich tyrosine kinase 2 inhibitor. Its function is to negate the activity of tyrosine 397 phosphorylation, subsequently diminishing cell growth, survival, and invasion. Chen et al. hypothesized that due to its mechanism of action, it may potentially also block mast cell activation and the resultant inflammatory response. Their preliminary results in the improvement of signs and symptoms in mice models with AC suggest that there is room for development of a therapeutic target in this area of drugs (Table 3) [190].

### 6.7. Biologics

Another potential therapeutic option is in the area of biologics. These agents work more specifically to block underlying inflammation [151]. Omalizumab has shown favorable results, particularly as a treatment for asthma that does not respond well to inhaled corticosteroids and is still under evaluation for AC. Due to its action on IgE-mediated conditions, subcutaneous administration of omalizumab is being investigated for the treatment of AC [191,192]. Omalizumab is a humanized monoclonal antibody that binds to FC portion of free floating IgE and has been shown in studies to reduce redness, tearing, and inflammation (Table 3). Though it is not yet approved for AC, studies thus far indicate success in treatment of AKC and VKC [193,194,195]. This drug appears promising and has consistently demonstrated that improved signs and symptoms of AC and quality of life with use [191,192].

Conversely however, dupilumab, which is an IL-4 and IL-13 pathway inhibitor, is reported to cause a higher rate of conjunctivitis and anterior segment inflammation as compared with placebo, though it has not been specifically studied in the context of AC treatment, nor have other biologics [196]. Dupilumab use for the treatment of AKC or VKC has not been effective at this time [197,198,199,200]. Other potential biologics that have yet to be evaluated for AC treatment are benralizumab, mepolizumab, and reslizumab [11,87].

### 6.8. Resolvins

Resolvins are lipid-derived molecules, specifically from polyunsaturated omega-3 fatty acid metabolism. They are biological, endogenous particles that are thought to be a component in the resolution of inflammation. There are two subtypes of resolvins, including those which are derived from eicosapentaenoic acid (EPA) omega-3’s or docosahexaenoic acid (DHA). RXE-10045 is an isopropyl ester synthetic derivative of EPA resolvins. This agent is currently under investigation for treatment of ocular inflammation, including AC, with potentially promising results in early phases (Table 3) [201,202].

### 6.9. Amniotic Stem Cells

Wu et al. hypothesized that there is a potential benefit from tissue stem cell conditioned medium (CM) on AC. In a study comparing various types of topically applied tissue stem cell CM, it was the human amniotic epithelial cell CM that demonstrated a significant reduction in the signs and symptoms of AC [203]. The utility in using tissue stem cells in general is their ability to self-renew and differentiate, thus allowing the repair of various types of damage. Furthermore, they possess immunomodulatory and anti-inflammatory properties [204,205,206,207]. Mesenchymal stem cells in particular have been documented to have a beneficial effect in other allergy-based conditions, including asthma, allergic rhinitis, rheumatoid arthritis, and allergic skin disease [207,208]. Human amniotic epithelial cells were also documented as a successful treatment of allergic airway disease [209]. The reason for these therapeutic effects is multifactorial and attributed to the inhibition of immune cells, such as effector T cells, effector B cells, neutrophils, dendritic cells, and mast cells, all of which are contributory to the exacerbation of the clinical expression of allergic disease (Table 3). Tissue stem cells may also secrete anti-inflammatory factors, such as neurotrophic factors, immunoregulatory cytokines, and chemokines. Specifically, these beneficial effects are dependent on interleukin-10, transforming growth factor-beta, indoleamine 2,3 dioxygenase (IDO), and prostaglandin E2, among others [210]. It is the abundance of some of these paracrine factors in amniotic epithelial stem cells specifically that was likely the reason it had the most effective treatment against AC. Additional characteristics are its ability to inhibit IgE release by effector B cells and mast cell activation and function, as well as exhibit an antihistamine effect. As such, data thus far supports continued research in this area as a potential, novel treatment for AC [203].

**Table 3:** Additional potential therapeutic targets in allergic conjunctivitis.

Non-Receptor Antagonists	Mechanism	Outcome
**Janus kinase inhibitor**	Blocks cytokine signaling and inhibits IL-2 mediated proliferation of T cells [171,172,173].	Significant improvement in the signs and symptoms of AC [176].
**Reactive aldehyde species (RASP) inhibitor**	Blocks pro-inflammatory cytokines and reduces histamine levels [179,180,181,182].	Prophylaxis and treatment of AC [183,184].
**Focal adhesion kinase (FAK) inhibitor**	Inhibits tyrosine 397 phosphorylation, thereby, preventing cell growth, survival, and migration [60,185,188,189].	Improvement in the signs and symptoms of allergic conjunctivitis [190].
**Other**	Mechanism	Outcome
**Omalizumab**	Binds to Fc region of free IgE to prevent IgE binding to receptors on mast cells [191,192,193,194,195].	Reduces redness, tearing, and inflammation [191,192].
**Resolvins**	Endogenous component in the resolution of inflammation [201,202].	Reducing signs and symptoms of AC [201,202].
**Amniotic stem cells**	Inhibition of effector T cells, effector B cells, neutrophils, dendritic cells, and mast cells and the secretion of anti-inflammatory cells [203,210].	Significant reduction in the signs and symptoms of AC [203].

IL-2: interleukin -2; AC: allergic conjunctivitis; Fc region: fragment crystallizable region; IgE: immunoglobulin E.

## 7. Novel Delivery Methods

While antihistamines, both oral and topical, have long been established as treatment for allergic disease and even AC, mizolastine is an up-and-coming drug. Possessing both antihistamine and anti-inflammatory properties, mizolastine is a benzimidazole derived selective H1 blocker, and differentiated from other drugs in its class because of its role in the inhibition of inflammatory cell chemotaxis and ICAM-1 expression [211]. A new mode for its delivery utilizing solid lipid nanoparticles has augmented its penetration onto the ocular surface, achieving a greater degree of bioavailability. In a study using rabbit eyes, El-Emam et al. concluded that mizolastine solid lipid nanoparticles reduced signs and symptoms of AC associated inflammation, including the expression of TNF-alpha and VEGF [212].

Pemirolast potassium is a mast cell stabilizer with proven efficacy in both the prophylaxis and treatment of AC [213,214,215]. In experimental animal models, the addition of levocabastine potentiates these effects [216]. These data suggests that ophthalmic use could potentially negate the effects of ocular allergy. However, pemirolast potassium is poorly absorbed by the ocular surface, which results in low bioavailability and little to no therapeutic benefit. A potential solution to this problem is the use of in situ gelling—a solution that transforms into a gel once administered at the treatment site. This process bypasses the challenges of topical ophthalmic drug administration, such as the normal pH, temperature, and ion content present within the normal tear film [217]. A recent study combining this optimal drug delivery system with pemirolast potassium yielded superior results as compared with traditional dosage forms, prompting the need for further research to achieve the most effective therapeutic result [218].

In 2019, Pall et al. conducted a large-scale study on antihistamine contact lens (CL) drug delivery system. A challenge often associated with topical treatment of AC is that the drops are not compatible with soft CL wearers. Using ketotifen in a soft CL to administer antiallergic treatment, patients in this study noticed significant improvement in itching, showing promise in this drug delivery system that would not only treat signs and symptoms of AC, but offer visual correction [219]. Similar findings were concluded using olopatadine HCl and epinastine HCl soft contact lens delivery models [220,221].

Mclaurin et al. studied the use of dexamethasone intracanalicular insert in AC and determined that, when compared to placebo, dexamethasone treated patients demonstrated significant reduction in ocular itching. Corticosteroids are often used with caution due to potentially harmful side effects, however in this study, only one patient (2.9%) exhibited intraocular pressure elevation. The remainder of adverse effects experienced were also low in the dexamethasone insert group, that mostly being tearing [222].

Additionally, immunomodulator tacrolimus has also been formulated in a study as a biodegradable, sustained-release microfilm drug delivery system. In mice models with AC, this system was as effective as topical eye drop administration, thus a potential option to successfully treat immune mediated ocular surface disease [223].

As demonstrated by these aforementioned examples, the drug delivery mode is as important as the type of drug. Particularly when it comes to topical, ocular administration for conjunctival disease, such as AC, the ocular surface is a challenging environment for sustained drug contact. A major limitation is bioavailability, which is why many ophthalmic drugs are administered through alternative, more invasive routes such as subconjunctival or intracameral injections. Emerging formulations utilizing nanotechnology may improve bioavailability by using nanoparticles to improve ocular penetration and reduce clearance. With regard to nanotechnology in AC, nano emulsions of tacrolimus and cyclosporine seem to improve bioavailability in early clinical trials [224,225]. The advantages to this method are many, and include sustained drug release, improved penetration, and retention onto the ocular surface. For ocular surface disease, such as AC, these characteristics are essential to ensure adequate drug delivery and therapeutic effect. It also aids with compliance, as the sustained and controlled release would reduce frequency of administration. A drawback, however, is that there is a higher likelihood of drug toxicity or negative effects. While there are many well delineated benefits to the use of nanotechnology, especially in the context of AC treatment, further trials are required to determine safe, non-toxic use [226].

## 8. Conclusions

As AC continues to become an increasingly prevalent condition with a significant impact on quality of life, the need for safe and effective treatment options is crucial [9,10,11,12]. While currently there are multiple management options available, a combination of therapies is often utilized to treat the signs and symptoms of AC. However, these combinations often offer only suboptimal relief in the signs and symptoms of AC, with the more potent treatment options posing a risk of harmful side effects. For decades, there have not been any new FDA approved agents developed in this area to remedy this problem. It is only through the precise understanding of the pathological and immune mechanisms involved in AC that therapeutic targets are able to be identified and formulated. At this time, many novel therapies and drug delivery modes discussed show evidence of future promise in the effective treatment of AC.

## Data Availability

No new data were created or analyzed in this study. Data sharing is not applicable to this article.
